# Radiosynthesis and Radiotracer Properties of a 7-(2-[^18^F]Fluoroethoxy)-6-methoxypyrrolidinylquinazoline for Imaging of Phosphodiesterase 10A with PET

**DOI:** 10.3390/ph5020169

**Published:** 2012-02-06

**Authors:** Uta Funke, Winnie Deuther-Conrad, Gregor Schwan, Aurélie Maisonial, Matthias Scheunemann, Steffen Fischer, Achim Hiller, Detlef Briel, Peter Brust

**Affiliations:** 1 Institute of Radiopharmacy, Research Site Leipzig, Helmholtz-Zentrum Dresden-Rossendorf, Permoserstraße 15, Leipzig 04318, Germany; Email: w.deuther-conrad@hzdr.de (W.D.-C.); aurelie.maisonial@inserm.fr (A.M.); m.scheunemann@hzdr.de (M.S.); s.fischer@hzdr.de (S.F.); a.hiller@hzdr.de (A.H.); p.brust@hzdr.de (P.B.); 2 Institute of Pharmacy, Universität Leipzig, Brüderstraße 34, Leipzig 04103, Germany; Email: gschwan@uni-leipzig.de (G.S.); briel@rz.uni-leipzig.de (D.B.)

**Keywords:** PDE10A, quinazoline, fluorine-18, positron emission tomography, PET

## Abstract

Phosphodiesterase 10A (PDE10A) is a key enzyme of intracellular signal transduction which is involved in the regulation of neurotransmission. The molecular imaging of PDE10A by PET is expected to allow a better understanding of physiological and pathological processes related to PDE10A expression and function in the brain. The aim of this study was to develop a new ^18^F-labeled PDE10A ligand based on a 6,7-dimethoxy-4-pyrrolidinylquinazoline and to evaluate its properties in biodistribution studies. Nucleophilic substitution of the 7-tosyloxy-analogue led to the 7-[^18^F]fluoroethoxy-derivative [^18^F]**IV** with radiochemical yields of 25% ± 9% (n = 9), high radiochemical purity of ≥99% and specific activities of 110–1,100 GBq/μmol. [^18^F]**IV** showed moderate PDE10A affinity (*K*_D,PDE10A_ = 14 nM) and high metabolic stability in the brain of female CD-1 mice, wherein the radioligand entered rapidly with a peak uptake of 2.3% ID/g in striatum at 5 min p.i. However, *ex vivo* autoradiographic and *in vivo* blocking studies revealed no target specific accumulation and demonstrated [^18^F]**IV** to be inapplicable for imaging PDE10A with PET.

## 1. Introduction

3'5'-cyclic nucleotide phosphodiesterases (PDEs) catalyze the hydrolysis of the secondary messengers adenosine (cAMP) and guanosine monophosphate (cGMP). Thus PDEs terminate intracellular signaling cascades which are related to various physiological processes such as immune responses, cardiac and smooth muscle contraction, visual response, ion channel conductance, apoptosis, and growth control [1,2]. Currently, 11 different families of PDEs are identified in mammals based on their primary amino acid and nucleotide sequences, regulatory properties, and substrate specificity [3,4,5] which hold great potential as drug targets for treatment of specific disease states [6].

The development of inhibitors of PDE10A, the only known member of the PDE10 family, for neurological and psychiatric disorders makes this enzyme an interesting target for molecular imaging approaches. Although studies on expression and pharmacological intervention suggest a relation between PDE10A and striatal hypofunction, the particular pathways which link PDE10A function and neurotransmission are not completely understood. In the brain, PDE10A shows the most unique distinctive expression pattern of all PDEs with particularly high mRNA levels in caudate nucleus and nucleus accumbens, and much lower levels in other CNS regions [7,8,9]. The graduation in transcript expression closely resembles the distribution of the enzyme assessed in detail by immunohistochemistry in different mammalian species [7,9,10]. Within the striatal area, PDE10A was shown to be highly expressed by the GABAergic medium spiny projection neurons which represent ~90% of striatal neurons [10]. The primary association of PDE10A with the post-synaptic membrane, indicated by subcellular fractionation, appears to link PDE10A activity with the regulation of excitability of medium spiny neurons [11]. Outside the brain, the level of PDE10A transcripts correlates with PDE10A immunoreactivity as well. Although high expression in developing spermatocytes, thyroid and pituitary gland as well as striated and cardiac muscle was detected [6,7,8,10], the physiological function of PDE10A in these peripheral organs is not known.

In accordance with the high expression of PDE10A in striatal regions, the systemic administration of papaverine, a potent PDE10A inhibitor, induced a rapid increase in striatal levels of cGMP and cAMP in mice [12] and demonstrated a broad-spectrum efficacy in a range of antipsychotic models [12,13]. Genetic deletion of PDE10A confirmed not only the involvement of this enzyme in the papaverine-mediated effects but has been reported to produce behavioral responses consistent with increased striatal output [14,15]. Furthermore, alterations in the levels of PDE10A transcripts have been related to synaptic plasticity in the hippocampus following longterm potentiation [16] and in animal models of neurodegenerative disorders [17].

These data form the basis for the hypothesis that PDE10A inhibitors will have potential for the treatment of neuropsychiatric disorders in humans that are correlated with striatal hypofunction such as schizophrenia, obsessive-compulsive disorders, Parkinson’s disease, and Huntington’s disease, and make PDE10A an interesting target for diagnostic and therapeutic monitoring by non-invasive imaging techniques such as positron emission tomography (PET). Therefore, numerous compounds which have been investigated concerning PDE10A inhibitory potency and behavioral impact [18,19,20,21,22] provide the current basis for the development of PDE10A targeting PET radiotracers.

**Figure 1:**
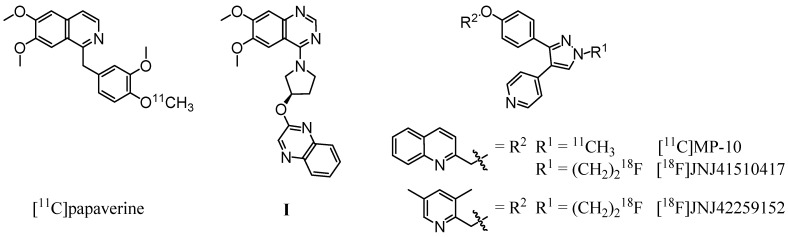
Radioligands investigated for PDE10A imaging and compound **I**, used as lead structure in this study.

Based on the PDE10A inhibitor papaverine (IC_50,PDE10A_ = 36 nM [12]), Tu *et al*. successfully synthesized a carbon-11 labeled derivative (Figure 1, left) by methylation of the corresponding 4-phenolate with [^11^C]methyl iodide [23]. Evaluation *in vivo* revealed an initially higher uptake in striatum in comparison to other brain regions. However, the very rapid clearance of the radioligand from the target region indicated [^11^C]papaverine as not suitable for imaging of PDE10A. A major progress was made by the same group [24] and Plisson *et al*. [25] with the development of a carbon-11 labeled analogue of the highly potent and selective PDE10A inhibitor MP-10 (IC_50,PDE10A_ = 1.26 nM [13], 0.37 nM [26]). Methylation of the *N*-desmethyl pyrazole precursor with [^11^C]methyl iodide led to [^11^C]MP-10 (Figure 1, right), showing a maximum striatum-to-cerebellum ratio of 6.55 in rats and 1.5 to 2 in rhesus monkey at 30 min post-injection (p.i.) [24]. PET studies of [^11^C]MP-10 in pig and baboon revealed a specific, discrete, and reversible uptake in striatum with a peak at 1–2 min p.i. in porcine and at 40–60 min p.i. in nonhuman primate brain [25].

Enabling high-resolution and long-term kinetic PET studies, ^18^F-labeled MP-10 derivatives were developed as well. (Figure 1, right). The *N*-[^18^F]fluoroethyl pyrazole derivative [^18^F]JNJ41510417 was synthesized by Celen *et al*. [27,28] using direct nucleophilic substitution of the corresponding *O*-mesylated precursor with n.c.a. [^18^F]fluoride (JNJ41510417 IC_50,PDE10A_ = 0.5 nM [28], pIC_50,PDE10A_ = 9.3 [29]). This radioligand showed reversible and PDE10A specific striatal binding in biodistribution as well as in dynamic small-animal PET studies with a maximum striatum-to-cerebellum ratio of 4.60 at 30 min p.i. [29]. Target specific binding was confirmed by imaging in PDE10A knockout mice. Nevertheless, high plasma protein binding of [^18^F]JNJ41510417, probably due to its high lipophilicity (JNJ41510417 c log *P* = 4.19 [29]), and detrimental slow kinetics have been observed [28]. This result prompted the same group to synthesize the smaller and less lipophilic dimethylpyridine analogue [^18^F]JNJ42259152 (JNJ42259152 c log *P* = 3.66, pIC_50,PDE10A_ = 8.8) [29,30] via alkylation of the *N*-desmethyl pyrazole precursor with [^18^F]fluoroethyl bromide. Biodistribution studies in rats demonstrated a striatum-to-cerebellum ratio of 5.38 at 30 min p.i. Dynamic small-animal PET imaging in rat and monkey showed highly intensive, reversible and PDE10A selective uptake of the radioligand in striatum, with low background activity in cortical regions and cerebellum [30]. In consequence, a first human study is planned for PDE10A imaging with [^18^F]JNJ42259152.

However, all MP-10 derived radioligands developed so far are adversely affected by metabolism [24,25,28,29,30]. Fast metabolism was observed in plasma of rats [28], baboon [24,25] and rhesus monkey [30]. The corresponding phenolic radioactive metabolite passed the blood-brain-barrier in rat and accumulated in the brain up to 15.9% [24]. In cerebellum up to 25.8% [28] and 53% [30] of metabolites were found at 60 min p.i.

As a structurally alternative approach and to possibly overcome the metabolic stability problem of the described PDE10A ligands, we intended to develop radioligands for PET imaging of PDE10A based on a 6,7-dimethoxyquinazoline (**I**, Figure 1, center). Published by a Pfizer research group in 2007, this potent papaverine-related ligand inhibited PDE10A with a *K*_i,PDE10A_ of 4 nM [31]. In this study we report on the development and investigation of a fluorine-18-labeled analogue. In a series of **I**-based compounds, the reference compound (*R*)-7-(2-fluoroethoxy)-6-methoxy-4-(3-(quinoxalin-2-yloxy)pyrrolidin-1-yl)quinazoline as well as two potential labeling precursors were synthesized in multi-step syntheses, and their PDE inhibitory potency was investigated [32] (data will be published in detail elsewhere). Since the 7-fluoroethoxyquinazoline derivative showed acceptable inhibitory potency with a *K*_i,PDE10A_ of 53 nM and occurred as radiochemically most accessible, this compound was chosen for radiolabeling with fluorine-18 and subsequent evaluation *in vitro* and *in vivo*.

## 2. Results and Discussion

### 2.1. Radiosyntheses

**Scheme 1:**
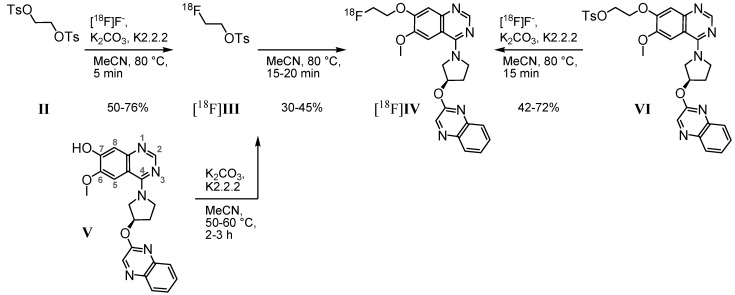
Two- and one-step radiofluorination for synthesis of [^18^F]**IV**.

Based on the 7-hydroxy precursor **V**, prepared via a 10-step synthesis [32], our first radiosynthetic approach to radioligand [^18^F]**IV** was carried out by applying 2-[^18^F]fluoroethyl-4-tosylate [^18^F]**III**(Scheme 1, left) as secondary labeling agent. According to Block *et al*., who described [^18^F]fluoroalkylation of *H*-acidic compounds [33,34], the tosylate **II** was substituted by n.c.a. [^18^F]fluoride via its K[^18^F]F-K2.2.2-carbonate complex. The resulting tosylate [^18^F]**III** was directly used for nucleophilic substitution by the phenolate **V**, previously activated under basic conditions.

**Figure 2:**
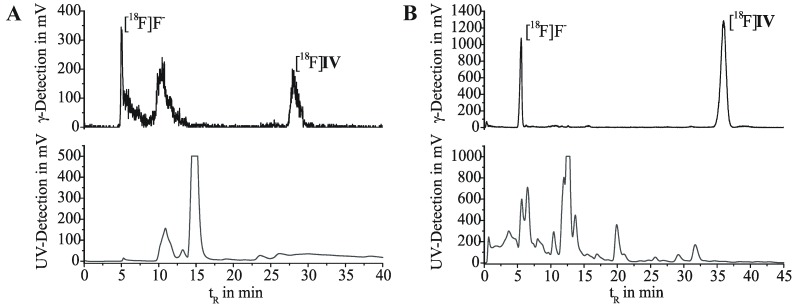
Semi-preparative HPLC chromatograms for purification of [^18^F]**IV**. (**A**) After two-step radiosynthesis. Multospher 120 RP-18 AQ column; 55% MeCN/H_2_O, 20 mM NH_4_OAc; flow rate 2 mL/min. (**B**) After one-step radiosynthesis. Reprosil-Pur C18-AQ column; 45% MeCN/H_2_O, 20mM NH_4_OAc; flow rate 1.75 mL/min.

First labeling as well as ^18^F-alkylation reactions were carried out in DMF at 110 °C. The reaction was accompanied by many side products and labeling efficiencies resulted in maximum 5% of [^18^F]**IV**. By changing the reaction medium to acetonitrile we achieved labeling efficiencies of 30%–45% for the ^18^F-alkylation step. Only a limited number of radioactive by-products occurred, according to radio-TLC and radio-HPLC analyses. However, the purification of [^18^F]**IV** was hindered due to lipophilic non-radioactive by-products. Even after solid phase extraction on alumina oxide and application of isocratic semi-preparative RP-HPLC (Figure 2A), UV-active contents could not be satisfactorily removed from the final product. As result for the two-step radiosyntheses, within 3.5 to 4.5 h for the entire process [^18^F]**IV** was obtained with radiochemical yields of 18%–29% (24% ± 6%, n = 4), but partially insufficient radiochemical purities of 92%–99% (95% ± 4%, n = 4).

According to this, either time consuming purification of the labeling agent [^18^F]**III**, or the more favorable development of a direct radiofluorination of a corresponding precursor was required. Thus, an 11-step synthesis provided the 7-(2-tosyloxyethyl) derivative **VI** [32]. Based on this, a one-step radiosynthesis of [^18^F]**IV** was established (Scheme 1, right). Most efficient nucleophilic ^18^F-for-OTs substitution was performed using the K[^18^F]F-K2.2.2-carbonate complex, determined by radio-TLC as well as analytical radio-HPLC. With 2–3 mg of precursor **VI** in acetonitrile after 15 min at 80 °C, labeling efficiencies of 42% to 72% of [^18^F]**IV** were observed. To reduce the basicity of the reaction mixture and to prevent tosylate decomposition, in an alternative approach K2.2.2 was replaced by tetrabutylammonium hydrogen carbonate as phase transfer catalyst (0.015 mmol, ABX GmbH, Radeberg, Germany; 0.5 mL acetonitrile). After 20 min at 80 °C the conversion of **VI** with n.c.a. [^18^F]fluoride resulted in [^18^F]**IV** with labeling efficiencies of 21%.

Purification of the crude [^18^F]**IV** was successfully carried out by solid phase extraction on a C-18 or silica gel cartridge by means of acetonitrile (methanol could be used as well), followed by isocratic elution on semi-preparative RP-HPLC (Figure 2B). Formulation of the final product for biological testing was done by solvent exchange on a C-18 cartridge, removal of the organic eluent (acetonitrile, ethanol or methanol) and dissolution in physiological saline. Following this process, we obtained the radioligand [^18^F]**IV** in radiochemical yields of 17%–40% (25% ± 9%, n = 10) within 3 to 4 h, with high chemical and radiochemical purities of ≥99%, and high specific activities in the range from 110 to 1,100 GBq/μmol.

### 2.2. Lipophilicity and Radioligand Stability *in Vitro*

To provide an indication of unspecific binding and blood-brain barrier permeability of [^18/19^F]**IV**, we calculated logarithmic distribution coefficients log *D*. The calculation was done with ACD/LogD (version 4.56, Advanced Chemistry Development Inc., Toronto, Canada) and MarvinSketch (ChemAxon Ltd., Budapest, Hungary). Values, given in Table 1, reached from a log *D*_7.2_ value of 2.27, which is, beside other criteria, pointing to an adequate brain penetration and optimum target to non-target ratio [35], up to log *D*_7.0–7.4_ = 3.52, indicating a high lipophilicity and possibly efficient brain uptake, but high unspecific binding as well.

**Table 1:** Calculated and experimentally determined logarithmic distribution coefficients of [^18/19^F]**IV**; values given in single, or mean ± SD with n ≥ 3.

Method	Specification	pH	Log *D*
Calculation	ACD/LogD	7.2	2.27
	MarvinSketch	7.0–7.4	3.52 ± 0.01
HPLC	Multospher, MeCN/NH_4_OAc	7.0	2.63 ± 0.01
	Supelcosil, MeCN/NH_4_OAc	7.0	2.63 ± 0.01
Shake flask	*n*-Octanol/phys. Phosphate	7.2	2.73 ± 0.59
	*n*-Octanol/Phosphate	7.2	2.64 ± 0.56
	*n*-Octanol/Tris-HCl	7.4	2.64 ± 0.55
	Average	7.2–7.4	2.67 ± 0.58

To clarify these findings, we determined the ligand distribution coefficients for [^18/19^F]**IV** experimentally, under physiological pH conditions in solid-liquid phase systems via retention on RP-HPLC, and in liquid-liquid phase systems by conventional shake-flask method. In three *n*-octanol/aqueous buffer solution systems at a pH range of 7.2 to 7.4, log *D* values of up to 2.73 were obtained for the radioligand (Table 1). With a retention time based capacity factor k in relation to those of standard substances [36], lipophilicity of **IV** was determined on two reversed phase/neutral buffered eluent systems with log *D* = 2.63. All in one, an experimental log *D*_7.0–7.4_ value of ~2.7 promised a good potential for brain uptake of [^18^F]**IV** *in vivo*, attended by low unspecific binding.

Furthermore, the chemical stability of [^18^F]**IV** *in vitro* under physiological conditions was investigated. After incubation at 40 °C for up to 1.5 h, the radioligand remained intact with ≥99% of native [^18^F]**IV** in 0.9% sodium chloride solution (pH 7.2) and in 0.01 M TRIS-HCl buffer (pH 7.4 at 21 °C), respectively, and showed a sufficient stability with 97% in physiological phosphate-saline (pH 7.2), verified by radio-TLC on silica gel and alumina oxide plates. For formulation and storage, radioligand stability in acetonitrile (98%, 1 h, 80 °C) and in ethanol (98%, 1 h, 80 °C; ≥99%, 1.5 h, 40 °C) was proven.

### 2.3. PDE10A Affinity and Selectivity

The dissociation constant *K*_D_ of [^18^F]**IV** was determined in homologous competitive binding experiments by nonlinear regression analysis [37]. Cell membrane homogenates from PDE10A-transfected SF21 cells (100 µg protein/mL incubation volume) were incubated with solutions of six concentrations of **IV** (working concentration 10 pM–10 µM), each spiked with a fixed concentration of [^18^F]**IV** (working concentration 0.05–0.1 nM). Based on an IC_50_ value of 39.3 (n = 2; 38.3 nM, 40.4 nM), a *K*_D_ of 13.8 nM (n = 2; 15.9 nM, 11.6 nM) was calculated.

Besides, inhibition of recombinant PDEs expressed in a baculovirus-SF21 cell system by measuring degradation of [^3^H]-cAMP revealed inhibitory potency of **IV** not only towards PDE10A (IC_50_ = 105 nM) but also towards PDE3A (IC_50_ = 88.7 nM) and PDE4A (IC_50_ = 562 nM), while the enzymatic activity of the PDEs 1B, 2A, 5A, 6, 7B, 8A, 9A, and 11A was not affected (IC_50_ > 1000 nM) [32]. Therefore, the resultant functional selectivity is moderate. Nevertheless, the distinctive expression of PDE10A with levels of mRNA much higher than that of PDE3A or PDE4A in striatum [8] was assumed to allow the detection of PDE10A expression by [^18^F]**IV**.

Contrary to the numerous investigations regarding the inhibitory potency of PDE10A ligands [18,19,20,21,22], so far no data have been published concerning the affinity of PDE10A inhibitors. Although the available data on PDE10A transcript and protein expression did not allow a pre-estimation of the *B*_max_ value of the target, with respect to the sub-nanomolar to nanomolar affinity values of successful PET-radioligands in neuroimaging [38], we assumed the nanomolar affinity of [^18^F]**IV** to be sufficient for specific visualization of PDE10A expression in the brain. However, an only recent report including data which allow the calculation of *B*_max _values of PDE10A very likely attenuates the imaging potential of [^18^F]**IV** due to an estimated *B*_max_ value of PDE10A binding sites of approximately 2 nM in the striatum [25].

**Figure 3:**
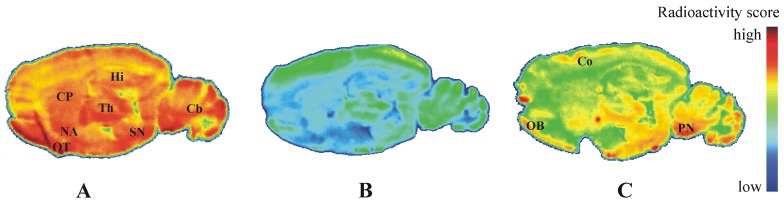
Representative color-coded autoradiographic images of sagittal rat brain slices. Distribution of radioactivity after 60 min of incubation with (**A**) 25 nM solution of [^18^F]**IV**, (**B**) 25 nM [^18^F]**IV**/10 μM **IV**, and (**C**) 25 nM [^18^F]**IV**/10 μM MP-10. CP, caudate putamen; NA, nucleus accumbens; OT, olfactory tubercle; Th, thalamus; Hi, hippocampus; SN, substantia nigra; Cb, cerebellum; Co, cortex; OB, olfactory bulb; PN, pontine nuclei.

### 2.4. Autoradiography *in Vitro*

*In vitro* co-incubation of rat brain slices with [^18^F]**IV** and **IV** revealed saturable binding of [^18^F]**IV** with an accumulation in PDE10A specific brain regions like caudate putamen, nucleus accumbens, substantia nigra, olfactory tubercle and cerebellum (Figure 3A,B). This pattern corresponds with the immunohistochemical localization of PDE10A in rat brain described in Seeger *et al*. [9].

However, [^18^F]**IV** binding was not completely inhibited by the highly PDE10A specific MP-10 (Figure 3C). In particular, non-displaceable binding of [^18^F]**IV** was observed in olfactory tubercle and cerebral regions. This result might indicate additional binding of [^18^F]**IV** to PDE3A, because of a non-negligible inhibitory potency of **IV** towards PDE3A (IC_50,PDE3A_ = 88.7 nM [32]) in association with a high expression of PDE3A mRNA in the olfactory bulb, cerebral cortex, cerebellum and the pontine nuclei of the rat brain, described by Reinhardt *et al*. [39]. Notably, Lakicz *et al*. [8] did not detect expression of PDE3A/B mRNA in human brain. Therefore, species-specific patterns of PDE expression have to be considered in the development of PDE10A targeting PET ligands based on the herein reported quinazoline structures. Furthermore, computer-assisted prediction of bioactivity of **IV** using Molinformation Cheminformatics software (Molinspiration Cheminformatics, Slovensky Grob, Slovak Republic) indicate high activity of compound **IV** as kinase inhibitor (score 0.92) and moderate activity as general enzyme inhibitor (score 0.57). Therefore, future work on structurally related PDE10 targeting ligands will have to focus on target selectivity in terms of kinase inhibitory potency.

### 2.5. Radioligand Stability *in Vivo*

**Figure 4:**
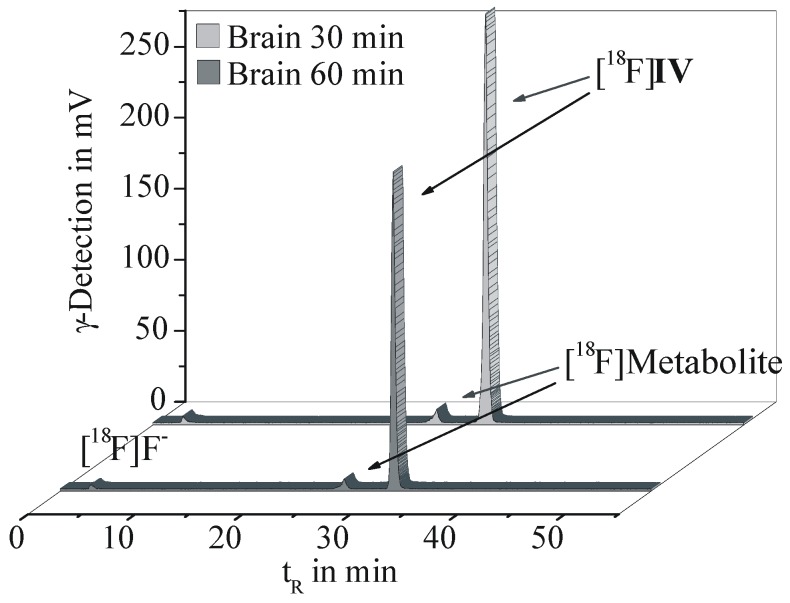
Analytical radio-HPLC results of brain homogenates obtained at 30 and 60 min p.i. of [^18^F]**IV** in female CD-1 mice (Reprosil Gold C18 column, gradient elution with 10 to 90% MeCN/H_2_O and 20 mM NH_4_OAc over 55 min, flow rate 1 mL/min).

The metabolic fate of [^18^F]**IV** was investigated in brain, plasma and urine samples of CD1-mice at 30 and 60 min p.i. Proteins were precipitated with acetonitrile and extraction efficiencies of 96.4% for brain homogenate, 90.2% for plasma and 89.4% for urine were obtained, indicating sufficient recovery rates. The supernatants of each material were analyzed by radio-TLC and analytical radio-HPLC, whereof two representative chromatograms are given in Figure 4.

In the brain, [^18^F]**IV** accounted for 94.3% and 93.1% of total radioactivity at 30 and 60 min p.i., respectively. A single lipophilic metabolite was detected representing only 4.2% of total radioactivity at 60 min p.i. Therefore a major impairment of the imaging properties of [^18^F]**IV** *in vivo* should not be expected. The same radiometabolite was detected in plasma with 23.5% and 22.0% of the total radioactivity at 30 and 60 min p.i., while the parent radiotracer [^18^F]**IV** accounted for 69.5% and 70.1%, respectively.

In urine the metabolic profile is distinctly different. While the non-metabolized [^18^F]**IV** and the single plasma- and brain-related radiometabolite accounted for only ~1% of the total radioactivity in urine, further radiometabolites were detected. The main three of them represent 11%, 12% and 44% of the total radioactivity. In contrast to brain and plasma samples, in urine free [^18^F]fluoride could be detected, although at a low level of only ~5% of total radioactivity.

### 2.6. Biodistribution and Specificity *in Vivo*

The organ distribution of [^18^F]**IV** after i.v. injection was investigated in female CD-1 mice. Time-activity data for ^18^F corresponding uptake in peripheral organs, in the brain and in particular in the striatum at 5, 30, 60 and 120 min p.i. are presented in Table 2.

**Table 2:** Biodistribution of [^18^F]**IV** in mice after injection of ~300 kBq without or with preadministration of 10 mg PDE10A-Inhibitor/kg.

	Control (% ID/g Wet Weight)				Blocking, 60 min p.i.	
Organ	5 min p.i. (n = 4)	30 min p.i. (n = 4)	60 min p.i. (n = 6)	120 min p.i. (n = 3)	MP-10 (n = 3)	I (n = 3)
Blood	1.88 ± 0.08	1.69 ± 0.38	1.45 ± 0.20	1.05 ± 0.10	1.85 ± 0.50	1.72 ± 0.16
Plasma	3.97 ± 1.41	2.87 ± 0.54	2.59 ± 0.36	2.13 ± 0.80	3.34 ± 1.00	2.70 ± 0.24
Brain	1.99 ± 0.21	1.54 ± 0.30	1.08 ± 0.26	0.77 ± 0.10	1.30 ± 0.20	1.13 ± 0.17
Striatum	2.32 ± 0.49	1.69 ± 0.44	1.14 ± 0.29	0.83 ± 0.10	1.38 ± 0.30	1.28 ± 0.27
Heart	4.01 ± 0.47	2.93 ± 0.71	2.16 ± 0.45	1.33 ± 0.20	2.89 ± 0.42	2.30 ± 0.22
Lungs	4.01 ± 0.51	3.51 ± 1.26	2.37 ± 0.62	1.46 ± 0.26	2.95 ± 0.86	2.62 ± 0.44
Stomach	6.14 ± 2.04	15.8 ± 6.09	12.6 ± 5.08	10.1 ± 5.96	14.6 ± 7.83	13.8 ± 1.65
Intestine	13.5 ± 4.15	43.2 ± 21.9	42.8 ± 33.5	49.3 ± 8.03	14.8 ± 4.16	44.3 ± 9.58
Colon	0.55 ± 0.05	0.88 ± 0.44	1.08 ± 0.43	3.49 ± 2.41	1.78 ± 0.78	1.07 ± 0.22
Liver	20.5 ± 1.87	16.4 ± 4.00	14.5 ± 4.54	7.05 ± 1.10	17.7 ± 3.87	12.9 ± 1.08
Kidneys	7.65 ± 0.54	5.34 ± 1.4	4.29 ± 0.93	2.13 ± 0.44	5.87 ± 1.56	4.01 ± 0.54
Urine	56.4 ± 105	18.5 ± 16.8	23.1 ± 9.31	30.9 ± 8.06	14.7 ± 7.17	18.0 ± 9.26
Bladder	1.85 ± 0.39	3.51 ± 1.39	2.60 ± 0.78	1.62 ± 0.28	2.93 ± 0.51	2.20 ± 0.49
Spleen	4.23 ± 0.51	2.85 ± 0.60	2.30 ± 0.25	1.21 ± 0.24	3.00 ± 0.79	2.48 ± 0.68
Thymus	3.10 ± 0.45	2.61 ± 0.50	2.08 ± 0.51	1.26 ± 0.07	2.55 ± 0.62	2.22 ± 0.08
Pancreas	5.19 ± 0.42	3.14 ± 0.70	2.77 ± 0.85	1.41 ± 0.07	3.19 ± 0.91	2.25 ± 0.26
Adrenals	25.4 ± 5.87	48.5 ± 16.2	28.2 ± 11.4	19.3 ± 13.2	36.0 ± 9.51	51.1 ± 17.9
Gonads	2.58 ± 0.46	3.94 ± 0.84	3.50 ± 1.22	1.64 ± 0.26	4.10 ± 1.41	3.30 ± 0.29
Muscles	1.75 ± 0.12	1.31 ± 0.32	1.22 ± 0.28	0.66 ± 0.01	1.29 ± 0.37	1.27 ± 0.25
Skin	n.d.	n.d.	1.45, 1.17 *	n.d.	2.43 ± 0.44	2.26 ± 0.82
Femurs	2.00 ± 0.36	2.14 ± 1.13	2.95 ± 3.73	1.61 ± 0.35	1.73 ± 0.58	1.62 ± 0.72
Femurs (fl.)	1.11 ± 0.32	0.67 ± 0.24	0.58 ± 0.42	1.15 ± 0.18	0.92 ± 0.23	0.81 ± 0.13

^18^F uptake values given in mean ± SD; n.d.: not determined. *: n = 2. fl.: flushed.

An initial brain uptake of ~2% ID/g was observed (brain: 1.99% ID/g; striatum: 2.32% ID/g) providing evidence for a moderate blood-brain barrier permeability of [^18^F]**IV**, as suggested by a lipophilicity value of log *D*_7.0–7.4_~2.7. However, striatum-to-blood ratios of 1.0 at 30 min p.i. and 0.8 at 60 and 120 min p.i. indicate insufficient uptake of [^18^F]**IV** in the mouse brain. This might be due to efflux mediated by multidrug resistance proteins expressed at the blood-brain barrier or due to insufficient target binding. A low uptake of radioactivity in femur of ~2% ID/g, which was reduced to about 1% ID/g after removal of highly lipophilic bone narrow, indicates negligible defluorination of the radioligand. The high uptake of radioactivity in urine with values of 56.4% and 30.9% ID/g at 5 and 120 min p.i., respectively, points to renal elimination as one major excretory pathway of [^18^F]**IV** and its metabolites, as already suggested by the metabolic studies discussed above. Furthermore, a major part of the radiotracer is excreted hepatobiliary, as indicated by the radioactivity uptake in liver (20.5% ID/g at 5 min p.i. to 7.05% ID/g at 120 min p.i.) and intestine (13.5% ID/g and 42.8% ID/g, 5 and 60 min p.i., respectively). The high accumulation of radioactivity in adrenals of up to 48.5% ID/g at 30 min p.i. points to the involvement of cytochrome P450 oxygenases in radiotracer metabolism, as these enzymes are highly expressed in adrenals [40].

The results of the blocking experiments, performed by pre-injection of the potent and selective PDE10A inhibitor MP-10 or of compound **I** indicated low specificity of [^18^F]**IV** binding in the brain and other tissues because no significant changes in the amount of accumulated radioactivity could be determined in all organs known to express PDE10A (*p* > 0.05). The observed slightly increase in the uptake of radioactivity in most organs might be caused by an increase in the free fraction of [^18^F]**IV** in plasma or changes in physiological parameters such as blood flow due to high concentrations of the blocking compounds.

### 2.7. Autoradiography *ex Vivo*

**Figure 5:**
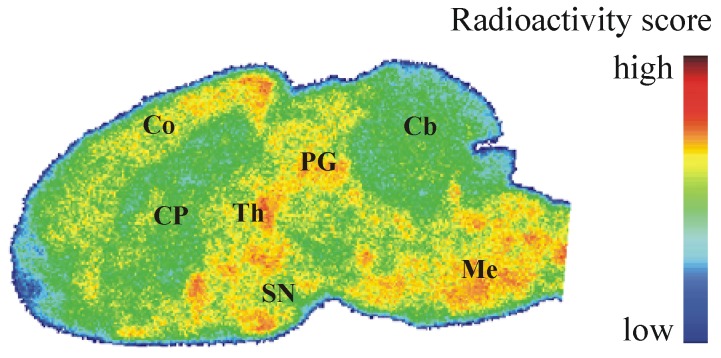
Representative color-coded autoradiographic image of a sagittal mouse brain slice. Distribution of [^18^F]**IV** in mice brain *ex vivo*, obtained at 30 min p.i. of 100 MBq. Co: cortex; CP: caudate putamen; PG: periaqueductal gray; Th: thalamus; SN: substantia nigra; Cb: cerebellum; Me: Medulla.

To determine the regional distribution of [^18^F]**IV** in the brain *in vivo*, we also performed *ex vivo* autoradiography. In CD-1 mice brain, heterogeneous distribution of radioactivity was noticed at 30 min after [^18^F]**IV** injection (Figure 5), but the localization of radioligand did neither correlate with the spatial distribution of PDE10A in the brain known from immunohistochemical [9] and *in vivo* PET studies [25] nor with the pattern of [^18^F]**IV** binding in the brain *in vitro* (Figure 3A). While increased radioligand binding was detected in cortex, periaqueductal gray, thalamus, substantia nigra and medulla, PDE10A-expressing brain regions such as caudate putamen, olfactory tubercle or cerebellum were free of significant accumulation of [^18^F]**IV** *in vivo*. The discrepancy in the pattern of [^18^F]**IV** distribution between *in vitro* and *ex vivo* autoradiography most probably indicates off-target binding of the radiotracer in combination with to low target affinity. While the dissociation rate underlying low affinity towards PDE10A allows a detection in the closed system of an *in vitro* study, the dynamic processes *in vivo* make binding of [^18^F]**IV** towards PDE10A non-detectable. Instead, the off-target binding of [^18^F]**IV** as already indicated by bioactivity score analyses has to be taken into account and will be considered in detail in future studies of potential PET ligands for PDE10A imaging. Altogether, the autoradiographic results verify the non-target binding of [^18^F]**IV** already indicated by the biodistribution studies and do not advocate the use of [^18^F]**IV** for *in vivo* imaging of PDE10A.

## 3. Experimental Section

### 3.1. General

No-carrier-added [^18^F]fluoride (half-life: 109.8 min) was produced via the [^18^O(p, n)^18^F] nuclear reaction by irradiation of a [^18^O]water target (>97% enriched, 2 mL) on a PETtrace cyclotron (16.5 MeV proton beam), GE Healthcare. The final radiolabeled product was purified by semi-preparative HPLC on a TRACERlab^TM^ FX F-N synthesizer (GE Healthcare, Waukesha, WI, USA) containing a S1021 pump (SYKAM Chromatographie Vertriebs GmbH, Fürstenfeldbruck, Germany); WellChrom K-2001 UV detector (KNAUER GmbH, Berlin, Germany); NaI(Tl)-counter; automated data acquisition, NINA, Nuclear Interface) and by solid phase extraction on SepPak^®^Plus cartridges (Waters Corporation, Milford, MA, USA). Analyses by TLC were performed on POLYGRAM® SIL G/UV254 and POLYGRAM^®^ ALOX N/UV254 plates, 40 × 80 mm (MACHEREY-NAGEL GmbH & Co. KG, Düren, Germany). Analytical HPLC was carried out on a computer assisted LC-2000*Plus* system from JASCO International Co., Ltd. (including LC-NetII/ADC Netbox, DG-2080-54 4-Line Degasser, LG-2080-04S quaternary gradient unit, PU-2080*Plus* HPLC Pump, AS-2055*Plus* Sampler, UV-2070*Plus* UV/Vis Detector), coupled with a radioactivity-HPLC-flow-monitor (Gabi Star, Raytest GmbH, Straubenhardt, Germany).

Processed TLC plates and organ sections were exposed to ^18^F-sensitive storage phosphor screens (BAS-TR2025, FujiFilm Co., Tokyo, Japan), and image plates were analyzed using a bioimaging analyzer system (BAS-1800 II, BASReader 2.26 Beta and AIDA 2.31 Image Analyzer software; Raytest GmbH, Straubenhardt, Germany, and FujiFilm Co., Tokyo, Japan).

*In vivo* studies were carried out in 3-months-old female CD-1 mice (20–25 g). Animals were obtained from the Medizinisch-Experimentelles Zentrum, Universität Leipzig, maintained on a 12 h light-dark cycle, and habituated for at least two days before experiments. All procedures involving animals were approved by the respective State Animal Care and Use Committee and conducted in accordance with the German Law for the Protection of Animals.

### 3.2. Radiochemistry

#### 3.2.1. Two-Step Radiosynthesis of (R)-7-(2-[^18^F]Fluoroethoxy)-6-methoxy-4-(3-(quinoxalin-2-yloxy)pyrrolidin-1-yl)quinazoline ([^18^F]**IV**)

A representative procedure is given as follows. For [^18^F]fluoride activation, colorless aqueous n.c.a. [^18^F]fluoride solution (1–2 mL) was added to 4,7,13,16,21,24-hexaoxa-1,10-diazabicyclo-[8.8.8]hexacosane (Kryptofix^®^, K2.2.2, 11.20 mg, 0.03 mmol), 0.15 M aqueous K_2_CO_3_ solution (89 μL, 0.013 mmol) and acetonitrile (1 mL) in a 10 mL conical vessel, equipped with magnetic stirrer, septum, as well as vacuum and inert gas access. This mixture was azeotropically dried according to previously described protocols [41,42], resulting in the K[^18^F]F-K2.2.2-carbonate complex, dissolved in anhydrous acetonitrile (1 mL). With 500 μL of the light yellow solution, nucleophilic substitution was performed in a 5 mL conical vessel under inert gas by reacting ethane-1,2-diyl bis(4-methylbenzenesulfonate) **II** (2.0 mg, 0.005 mmol, Scheme 1) under stirring at 80 °C, using a heating block. To monitor the fluorination step, 15 μL aliquots were taken, diluted and analyzed (radio-HPLC, isocratic elution with 45% MeCN/H_2_O and 15 mM AcOH/12.5 mM TEA, flow rate 1 mL/min, λ = 245 nm; Kromasil 100 C18, 5 μm, 250 × 4.6 mm, JASCO International Co., Ltd., t_R,[18F]**III**_ = 15 min; radio-TLC, SiO_2_ with petroleum ether/EtOAc/NH_3_aq. 25%, 5/5/0.25, v/v/v, R_f,[18F]**III**_ = 0.72). Thus, a labeling efficiency of 75% was observed after 5 min of reaction.

In a 2 mL reaction vessel, equipped with magnetic stirrer, septum and inert gas access, (*R*)-7-hydroxy-6-methoxy-4-[3-(quinoxalin-2-yloxy)pyrrolidine-1-yl]quinazoline (**V**, 4.0 mg, 0.01 mmol) in acetonitrile (1 mL) were stirred with Kryptofix^®^ (1.90 mg, 0.01 mmol) and K_2_CO_3_ (0.35 mg, 0.005 mmol) at 60 °C, using a heating block. After 2 h, the acetonitrile was carefully evaporated to dryness under inert gas and the mixture dissolved in anhydrous acetonitrile (1 mL). At room temperature, the yellow phenolate-containing solution (500 μL, 0.005 mmol) was added to a solution of [^18^F]**III** (500 μL) in a conical 6 mL vessel and stirred under inert gas at 80 °C, using a heating block. For reaction control, aliquots of 15 µL were taken, diluted and analyzed on radio-TLC (SiO_2_ with MTBE/MeOH/NH_3_aq. 25%, 10/1/0.25, v/v/v, R_f,[18F]**IV**_ = 0.45), as well as radio-HPLC (isocratic elution with 45% MeCN/H_2_O and 15 mM AcOH/12.5 mM TEA, flow rate 1 mL/min, λ = 245 nm; Kromasil 100 C18, 5 μm, 250 × 4.6 mm, JASCO International Co., Ltd., t_R,[18F]**IV**_ = 12 min). After 15 min reaction time, labeling efficiencies of ~38% were observed.

The crude, ochre-coloured reaction mixture was cooled to room temperature, diluted with water (50 mL) and passed through a neutral Al_2_O_3_ cartridge. The crude product [^18^F]**IV** was eluted with acetonitrile (1.5 mL), diluted to 4 mL with water and subjected to semi-preparative radio-HPLC (isocratic elution with 55% MeCN/H_2_O, 20 mM NH_4_OAc; λ = 254 nm; flow rate 2 mL/min on a RP-column with precolumn, 50 × 10 mm and 150 × 10 mm, Multospher 120 RP-18 AQ, CS-Chromatographie Service GmbH, Langerwehe, Germany; t_R,[18F]**IV**_ = 28 min). Fractions containing [^18^F]**IV** were collected and analyzed on radio-TLC as well as radio-HPLC. After 3.5 h for the entire process, the final product was obtained with a decay corrected radiochemical yield of 29% (based on cyclotron produced [^18^F]F^−^) and a radiochemical purity of 99%, maintaining unspecified chemical impurities.

#### 3.2.2. One-Step Radiosynthesis of [^18^F]**IV**

A representative procedure is given as follows. The K[^18^F]F-K2.2.2-carbonate complex was prepared as described in Section 3.2.1. and dissolved in anhydrous acetonitrile (500 μL). Under inert gas, the light yellow solution (250 μL) was added to (*R*)-2-(6-methoxy-4-(3-(quinoxalin-2-yloxy)pyrrolidin-1-yl)quinazolin-7-yloxy)ethyl 4-methylbenzenesulfonate (**VI**, 2.1 mg, 0.004 mmol, Scheme 1), dissolved in acetonitrile (250 μL), and stirred at 80 °C, using a heating block. Reaction monitoring was done by taking 15 μL aliquots which were diluted and analyzed on radio-TLC (SiO_2_ with MTBE/MeOH/NH_3_aq. 25%, 9/1/0.25, v/v/v, R_f,[18F]**IV**_ = 0.72; Al_2_O_3_ with cyclohexane/EtOAc, 1/9, v/v, R_f,[18F]**IV**_ = 0.63), as well as radio-HPLC (gradient elution with 10 to 90% MeCN/H_2_O and 20 mM NH_4_OAc over 55 min, flow rate 1 mL/min, λ = 245 nm; Reprosil Gold C18, 5 μ, 250 × 4.6 mm, Maisch HPLC GmbH, Ammerbuch-Entringen, Germany, t_R,[18F]**IV**_ = 31 min). Thus, 10 min of reaction led to a labeling efficiency of 60%.

At room temperature, the ochre-coloured reaction mixture was diluted with water (50 mL) and passed through a SiO_2_ or C-18 cartridge, and crude [^18^F]**IV** was eluted with acetonitrile (1.25 mL). After dilution to 4 mL with water, the cloudy solution was manually injected to a semi-preparative radio-HPLC. During isocratic elution (50% MeCN/H_2_O, 20 mM NH_4_OAc; λ = 254 nm; flow rate 1.5 mL/min on a RP-column with precolumn, 50 × 10 mm and 150 × 10 mm, Reprosil-Pur C18-AQ, 7 μ, Maisch HPLC GmbH, Ammerbuch-Entringen, Germany; t_R,[18F]**IV**_ = 32 min), fractions containing [^18^F]**IV** were collected and analyzed with radio-TLC, as well as radio-HPLC (see above). The combined fractions were diluted with water (40 mL) and passed through a C-18 cartridge. After washing with water (2 mL), [^18^F]**IV** was eluted with ethanol (1.25 mL) and the solvent was carefully evaporated under argon. The desired radiotracer was then dissolved in a solution of 5% ethanol in a 0.9% sodium chloride isotonic solution. Within 3.3 h for the entire process, [^18^F]**IV** was obtained in a decay corrected radiochemical yield of 40% (based on cyclotron produced [^18^F]F^−^), a chemical and radiochemical purity of ≥99%, and specific activity of 269 GBq/μmol (determination via analytical HPLC with UV/mass calibration). The final product [^18^F]**IV** was shown by radio-TLC (see above) and analytical radio-HPLC (gradient elution with 10% to 90% MeCN/H_2_O and 20 mM NH_4_OAc over 80 min, flow rate 1 mL/min, λ = 245 nm; Reprosil Gold C18, 5 μ, 250 × 4.6 mm, Maisch HPLC GmbH, Ammerbuch-Entringen, Germany, t_R,[18F]**IV**_ = 39 min) to be identical to the authentic non-radioactive material **IV** [32] and to be free of significant chemical and radiochemical impurities.

### 3.3. Determination of Lipophilicity of [^18/19^F]IV

#### 3.3.1. Determination of Log D_7.2–7.4_ Values by Shake-Flask Method

Log *D*_7.2–7.4_ values of [^18^F]**IV** were determined using a conventional shake-flask method (multiple distribution in triplicates). Measurements of distribution coefficients were performed in 3 series at 21 °C between *n*-octanol and three aqueous buffer solutions, physiological phosphate-saline (0.1 M Na_3_PO_4_, 0.15 M NaCl, pH 7.2), phosphate buffer (KH_2_PO_4_/Na_2_HPO_4_•2H_2_O, 50 mM, pH 7.2) or TRIS-HCl buffer (50 mM, pH 7.4 at 21 °C). Solutions of [^18^F]**IV** (2–5 MBq) in MeCN, MeOH (100 μL) or in 5% ethanol/0.9% NaCl (10 μL) were added to a 15 mL polypropylene tube (n = 3 per buffer system) containing a two-layer system of a selected aqueous solution (3 mL) and *n*-octanol (3 mL). Both phases were presaturated with the corresponding aqueous solution or *n*-octanol, respectively. The tubes were shaken for 30 min (200 rpm, HS250 basic, IKA^®^ Labortechnik GmbH & Co. KG, Staufen, Germany) and then centrifuged at 6000 rpm (21 °C, 5 min; EBA 12R, Andreas Hettich GmbH & Co. KG, Tuttlingen, Germany). Aliquots of 1 mL of the *n*-octanol phase and 1 mL of the aqueous phase were transferred into separate sample tubes with care. Radioactivity of these samples was quantified using a calibrated γ-counter (Wallac WIZARD, Perkin Elmer, Waltham, MA, USA). The procedure mentioned above was repeated for each serial 3–4 times using 1 mL of the *n*-octanol phase collected after each extraction step. Distribution coefficients and log *D*_7.2–7.4_ values of [^18^F]**IV** were calculated with Microcal^TM^ Origin (6.0, Microcal Software, Inc., Northhampton, MA, USA).

#### 3.3.2. Determination of log D_7.0_ Values by RP-HPLC Retention

Referring to an OECD guideline [36] and using a series of reference compounds (nitrobenzene, *p*-nitrophenol, benzene, toluene, chlorobenzene, benzophenone, naphthalene, 1,4-dibromobenzene, biphenyl, diphenylether, 1,4-diiodobenzene) with known log *P*_OW_ values comprised between 1.89 and 4.64, retention times were determined on two reversed phase columns (Multospher 120 RP-18 AQ, 250 mm × 4.6 mm, 5 μm, CS-Chromatographie Service GmbH, Langerwehe, Germany; SupelcosilTM ABZ + Plus, 250 × 4 mm, 5 μm, Supelco, Bellefonte, PA, USA). On an 1100 HPLC ChemStation (Agilent Technologies, Santa Clara, CA, USA) using a gradient (5% to 80% MeCN/H_2_O and 20 mM NH_4_OAc over 65 min, pH = 7.0, flow rate 1 mL/min, λ = 254 nm), reference compound **IV** (t_R,**IV**@Multospher_ = 43.7, t_R,**IV**@Supelcosil_ = 27.9 min) as well as standard compounds have been eluted with n ≥ 3 on each column. Dead times t_0_ were determined by retention of thiourea. Corresponding capacity factors k for all compounds were calculated and plotted as log k *versus* log *P* of the standard compounds, affording calibration curves (Microcal^TM^ Origin 6.0, Microcal Software, Inc., Northhampton, MA, USA). The regression equations were then used to calculate the log *D*_7.0_ values of **IV** via its corresponding log k (values are given in Table 1).

### 3.4. *In Vitro* Characterization

#### 3.4.1. PDE10A Affinity

Crude membrane homogenates isolated from PDE10A-transfected SF21 cells were obtained from Biocrea GmbH, Radebeul, Germany [43]. For competitive radioligand displacement experiments (n = 2), the membrane preparations were diluted with incubation buffer (50 mM Tris-HCl, pH 7.4 at room temperature, 5 mM MgCl_2_) and re-homogenized by passing through a 27-gauge needle. The protein (100 µg) was incubated with 0.05–0.1 nM of [^18^F]**IV** and different concentrations of **IV** (10 pM–1 µM), diluted from a 10 mM stock in DMSO, at a final volume of 1 mL. Nonspecific binding was determined in the presence of 10 µM of **I**, and control incubation was carried out accordingly using crude membrane preparations obtained from non-transfected SF21 cells.

Incubation at room temperature was terminated after 120 min by rapid filtration (Brandel 48-channel harvester; Biomedical Research and Development Lab. Inc., Gaithersburg, MD, USA) on Whatman GF/B glass-fiber filters, pre-soaked for 90 min in 0.3% polyethylenimine (Sigma, Deisenhofen, Germany), followed by washing four times with 4 mL of ice-cold wash buffer (50 mM Tris-HCl, pH 7.4 at 4 °C). Filter-bound radioactivity was quantified using a calibrated γ-counter (Wallac WIZARD, Perkin Elmer, Waltham, MA, USA).

#### 3.4.2. Data Analysis

Each experiment was carried out in triplicate. The binding parameter *IC*_50_ was estimated using iterative non-linear curve fitting. *K*_D_ of [^18^F]**IV** was estimated directly from homologous competition curve with *K*_D_ = IC_50_-[radioligand] as well as from the respective saturation isotherm by nonlinear regression analysis [37].

#### 3.4.3. *In Vitro* Autoradiographic Study

Frozen sagittal brain sections (12 μm) obtained from female SPRD rats (8–10 weeks) were thawed, dried in a stream of cold air, preincubated for 20 min with 50 mM TRIS-HCl, pH 7.4, at room temperature, and dried again. For determination of total binding, sections were incubated with a 25 nM solution of [^18^F]**IV** in 50 mM TRIS-HCl, pH 7.4, at room temperature for 60 min. Nonspecific binding was determined in the presence of 100, 10, and 1 µM of **IV** or MP-10 [13,26] (MP-10 obtained as a gift from Bjarke Ebert, H. Lundbeck A/S, Valby Denmark). After incubation, sections were washed twice for 2 min with 50 mM TRIS-HCl, pH 7.4 at 4 °C, dipped briefly in ice-cold deionized water (5 s), dried in a stream of cold air, and exposed for 17 h to ^18^F-sensitive storage phosphor screens, which were analyzed using the BAS-1800 II system (see above).

### 3.5. Biodistribution and Metabolism Studies in Mice

#### 3.5.1. Metabolic Stability

After injection via the tail vein of ~150 MBq of [^18^F]**IV** in 200 mL isotonic solution, mice were sacrificed at 30 or 60 min p.i. (n = 2 per time). Urine and blood samples were obtained, and whole brains were isolated and homogenized in 50 mM TRIS-HCl, pH 7.4/4 °C, in a borosilicate glass cylinder by 10 strokes of a PTFE plunge at a speed of 1,000 rpm using a Potter S Homogenizer (B. Braun Melsungen AG, Germany). Plasma samples were obtained by centrifugation of blood at 2,000 g and 4 °C for 10 min. Extractions of brain homogenate, plasma and urine samples were done by treatment with ice-old acetonitrile (1:5 v/v; 2 min vortex mixing, Vortex-Genie 2, Scientific Industries Inc., NY, USA; 10 min incubation on ice) and centrifugation (6,000 rpm, 4 °C, 5 min; EBA 12R, Andreas Hettich GmbH & Co. KG, Tuttlingen, Germany). The supernatants were stored separately. Sediments of brain and plasma samples were re-suspended in 500 μL ice-cold acetonitrile and extraction was repeated. Supernatants of each specimen were combined and slowly evaporated at 70 °C under nitrogen stream. Analysis of radioactive content of the supernatant fractions resolved in 50% MeCN/H_2_O and 20 mM NH_4_OAc was carried out by analytical radio-HPLC (gradient elution with 10 to 90% MeCN/H_2_O and 20 mM NH_4_OAc over 55 min, flow rate 1 mL/min, λ = 245 nm; Reprosil Gold C18, 5 μ, 250 × 4.6 mm, Maisch HPLC GmbH, Ammerbuch-Entringen, Germany) and radio-TLC (SiO_2_ with MTBE/MeOH/NH_3_ aq. 25%, 9/1/0.25, v/v/v; Al_2_O_3_ with Cyclohexane/EtOAc/NH_3_ aq. 25%, 1/9/0.25, v/v/v), processed by radioluminescence imaging (see above). Extraction rates were estimated by measuring aliquots of supernatant and precipitate samples using a calibrated γ-counter (Wallac WIZARD, Perkin Elmer, Waltham, MA, USA).

#### 3.5.2. Biodistribution and Regional Brain Uptake Studies

Conscious mice received a tail-vein injection of about 300 kBq of [^18^F]**IV**, dissolved in 200 µL isotonic saline. At 5, 30, 60 and 120 min p.i. animals (n = 3–6 per time) were anaesthetized, and blood and urine samples were taken. After sacrifice, organs of interest were removed, separated, weighed, and radioactivity was measured in a calibrated γ-counter (Wallac WIZARD, Perkin Elmer, Waltham, MA, USA). Striatum was dissected from the brain and investigated separately. The percentage of the injected dose per gram of wet tissue (% ID/g) was calculated by the quotient of tissue counts and initial dose counts, and weight of the tissue sample.

Specificity of brain and in particular striatal uptake of [^18^F]**IV** was investigated in blocking studies by intraperitoneal injection of 10 mg/kg of **I** [31] (n = 3) or 10 mg/kg of MP-10 [13,26] (n = 3), dissolved in 200 µL saline at 10 min before the radiotracer. Mice were sacrificed at 60 min p.i., and the radioligand uptake was determined as described above. Data listed in table 2 are given as mean values ± standard deviation (SD).

#### 3.5.3. *Ex Vivo* Autoradiographic Study

*Ex vivo* autoradiography on the distribution of [^18^F]**IV** in mouse brain (n = 1) was performed on sagittal brain sections obtained at 30 min after i.v. injection of 100 MBq of [^18^F]**IV**. After sacrifice, the brain was removed and dissected hemispheres were frozen in isopentane (−35 °C). Sagittal sections (12 µm) were cut on a cryostat microtome (Microm, Walldorf, Germany), mounted onto microscope slides and dried in a stream of cold air for 10 min. After exposition to ^18^F-sensitive storage phosphor screens for 60 min, image plates were analyzed using a bioimaging analyzer system (see above).

## 4. Conclusions

In our efforts to develop a PET ligand for imaging PDE10A *in vivo* based on a known structure **I**, we successfully synthesized a first [^18^F]fluoroalkyl-substituted pyrrolidinylquinazoline [^18^F]**IV** with satisfying radiochemical yield, and of high radiochemical purity as well as specific activity. [^18^F]**IV** possesses a high chemical stability and a lipophilicity suitable for a sufficient passage of the blood-brain barrier, shows moderate PDE10A affinity and target specific binding in the brain *in vitro*, and demonstrates fast initial uptake as well as high metabolic stability in mice brain. However, the uptake of [^18^F]**IV** in the brain of mice and in particular in the PDE10A-rich striatum could neither be blocked by co-administration of **I** nor by the highly PDE10A-specific MP-10. Furthermore, the pattern of radiotracer accumulation in the brain does not correspond to the unique distribution of PDE10A protein. Besides a target affinity of *K*_D_ ~ 14 nM, insufficient to visualize endogenous PDE10A expression with an estimated *B*_max_ of ~2 nM, non-negligible off-target binding of [^18^F]**IV** does not allow the detection of PDE10A expression patterns and quantities.

In summary, despite superior metabolic properties of [^18^F]**IV** in comparison with MP-10 related PDE10A radiotracers, findings obtained in animal experiments make the 7-[^18^F]fluoroethoxy-6-methoxy-4-pyrrolidinylquinazoline [^18^F]**IV** unsuitable for quantitative imaging of PDE10A. Further developments of PET ligands for PDE10A imaging should be based on an alternative structural approach.

## References

[1] Beavo J.A. (1995). Cyclic nucleotide phosphodiesterases: Functional implications of multiple isoforms. Physiol. Rev..

[2] Francis S.H., Turko I.V., Corbin J.D. (2001). Cyclic nucleotide phosphodiesterases: Relating structure and function. Prog. Nucleic Acid Res. Mol. Biol..

[3] Keravis T., Lugnier C. (2010). Cyclic nucleotide phosphodiesterases (PDE) and peptide motifs. Curr. Pharm. Des..

[4] Lugnier C. (2006). Cyclic nucleotide phosphodiesterase (PDE) superfamily: A new target for the development of specific therapeutic agents. Pharmacol. Ther..

[5] Surapisitchat J., Beavo J.A., Dennis E.A., Bradshaw R.A. (2011). Phosphodiesterase families. Transduction Mechanisms in Cellular Signaling: Cell Signaling Collection.

[6] Bender A.T., Beavo J.A. (2006). Cyclic nucleotide phosphodiesterases: Molecular regulation to clinical use. Pharmacol. Rev..

[7] Fujishige K., Kotera J., Omori K. (1999). Striatum- and testis-specific phosphodiesterase PDE10A. Isolation and characterization of a rat PDE10A. Eur. J. Biochem..

[8] Lakics V., Karran E.H., Boess F.G. (2010). Quantitative comparison of phosphodiesterase mRNA distribution in human brain and peripheral tissues. Neuropharmacology.

[9] Seeger T.F., Bartlett B., Coskran T.M., Culp J.S., James L.C., Krull D.L., Lanfear J., Ryan A.M., Schmidt C.J., Strick C.A. (2003). Immunohistochemical localization of PDE10A in the rat brain. Brain Res..

[10] Coskran T.M., Morton D., Menniti F.S., Adamowicz W.O., Kleiman R.J., Ryan A.M., Strick C.A., Schmidt C.J., Stephenson D.T. (2006). Immunohistochemical localization of phosphodiesterase 10A in multiple mammalian species. J. Histochem. Cytochem..

[11] Xie Z., Adamowicz W.O., Eldred W.D., Jakowski A.B., Kleiman R.J., Morton D.G., Stephenson D.T., Strick C.A., Williams R.D., Menniti F.S. (2006). Cellular and subcellular localization of PDE10A, a striatum-enriched phosphodiesterase. Neuroscience.

[12] Siuciak J.A., Chapin D.S., Harms J.F., Lebel L.A., McCarthy S.A., Chambers L., Shrikhande A., Wong S., Menniti F.S., Schmidt C.J. (2006). Inhibition of the striatum-enriched phosphodiesterase PDE10A: A novel approach to the treatment of psychosis. Neuropharmacology.

[13] Grauer S.M., Pulito V.L., Navarra R.L., Kelly M.P., Kelley C., Graf R., Langen B., Logue S., Brennan J., Jiang L. (2009). Phosphodiesterase 10A inhibitor activity in preclinical models of the positive, cognitive, and negative symptoms of schizophrenia. J. Pharmacol. Exp. Ther..

[14] Siuciak J.A., McCarthy S.A., Chapin D.S., Martin A.N., Harms J.F., Schmidt C.J. (2008). Behavioral characterization of mice deficient in the phosphodiesterase-10A (PDE10A) enzyme on a C57/Bl6N congenic background. Neuropharmacology.

[15] Siuciak J.A., McCarthy S.A., Chapin D.S., Fujiwara R.A., James L.C., Williams R.D., Stock J.L., McNeish J.D., Strick C.A., Menniti F.S. (2006). Genetic deletion of the striatum-enriched phosphodiesterase PDE10A: Evidence for altered striatal function. Neuropharmacology.

[16] O'Connor V., Genin A., Davis S., Karishma K.K., Doyère V., de Zeeuw C.I., Sanger G., Hunt S.P., Richter-Levin G., Mallet J. (2004). Differential amplification of intron-containing transcripts reveals long term potentiation-associated up-regulation of specific Pde10A phosphodiesterase splice variants. J. Biol. Chem..

[17] Hu H., McCaw E.A., Hebb A.L.O., Gomez G.T., Denovan-Wright E.M. (2004). Mutant huntingtin affects the rate of transcription of striatum-specific isoforms of phosphodiesterase 10A. Eur. J. Neurosci..

[18] Siuciak J.A. (2008). The role of phosphodiesterases in schizophrenia: Therapeutic implications. CNS Drugs.

[19] Siuciak J.A., Strick C.A. (2007). Phosphodiesterase 10A inhibitors as a novel therapeutic approach for schizophrenia. Expert Opin. Drug Discov..

[20] Kehler J., Ritzén A., Greve D.R. (2007). The potential therapeutic use of phosphodiesterase 10 inhibitors. Expert Opin. Ther. Pat..

[21] Kehler J., Kilburn J.P. (2009). Patented PDE10A inhibitors: Novel compounds since 2007. Expert Opin. Ther. Pat..

[22] Kehler J., Nielsen J. (2011). PDE10A inhibitors: Novel therapeutic drugs for schizophrenia. Curr. Pharm. Des..

[23] Tu Z., Xu J., Jones L.A., Li S., Mach R.H. (2010). Carbon-11 labeled papaverine as a PET tracer for imaging PDE10A: Radiosynthesis, *in vitro* and *in vivo* evaluation. Nucl. Med. Biol..

[24] Tu Z., Fan J., Li S., Jones L.A., Cui J., Padakanti P.K., Xu J., Zeng D., Shoghi K.I., Perlmutter J.S., Mach R.H. (2011). Radiosynthesis and *in vivo* evaluation of [^11^C]MP-10 as a PET probe for imaging PDE10A in rodent and non-human primate brain. Bioorg. Med. Chem..

[25] Plisson C., Salinas C., Weinzimmer D., Labaree D., Lin S.-F., Ding Y.-S., Jakobsen S., Smith P.W., Eiji K., Carson R.E. (2011). Radiosynthesis and *in vivo* evaluation of [^11^C]MP-10 as a positron emission tomography radioligand for phosphodiesterase 10A. Nucl. Med. Biol..

[26] Verhoest P.R., Chapin D.S., Corman M., Fonseca K., Harms J.F., Hou X., Marr E.S., Menniti F.S., Nelson F., O'Connor R. (2009). Discovery of a novel class of phosphodiesterase 10A inhibitors and identification of clinical candidate 2-[4-(1-methyl-4-pyridin-4-yl-1*H*-pyrazol-3-yl)-phenoxymethyl]-quinoline (PF-2545920) for the treatment of schizophrenia. J. Med. Chem..

[27] Celen S., Angelis M.D., Chitneni S.K., Alcazar J., Koole M., Dedeurwaerdere S., Steckler T., Schmidt M., Laere K.V., Verbruggen A. (2009). Synthesis and preliminary biological evaluation of [^18^F]JNJ41510417 as a radioligand for positron emission tomography imaging of phosphodiesterase-10A in the brain. Eur. J. Nucl. Med. Mol. Imaging.

[28] Celen S., Koole M., Angelis M.D., Sannen I., Chitneni S.K., Alcazar J., Dedeurwaerdere S., Moechars D., Schmidt M., Verbruggen A. (2010). Preclinical evaluation of ^18^F-JNJ41510417 as a radioligand for PET imaging of phosphodiesterase-10A in the brain. J. Nucl. Med..

[29] Andrés J.-I., de Angelis M., Alcázar J., Iturrino L., Langlois X., Dedeurwaerdere S., Lenaerts I., Vanhoof G., Celen S., Bormans G. (2011). Synthesis, *in vivo* occupancy, and radiolabeling of potent phosphodiesterase subtype-10 inhibitors as candidates for positron emission tomography imaging. J. Med. Chem..

[30] Celen S., de Angelis M., Koole M., Alcazar J., Sannen I., Cornelis J., Dedeurwaerdere S., Schmidt M., van Laere K., Verbruggen A. (2011). [^18^F]JNJ42259152 as potential radioligand for positron emission tomography imaging of phosphodiesterase-10A in the brain. J. Labelled Comp. Radiopharm..

[31] Chappie T.A., Humphrey J.M., Allen M.P., Estep K.G., Fox C.B., Lebel L.A., Liras S., Marr E.S., Menniti F.S., Pandit J. (2007). Discovery of a Series of 6,7-Dimethoxy-4-pyrrolidylquinazoline PDE10A inhibitors. J. Med. Chem..

[32] Nieber K., Erdmann S., Briel D., Schwan G., Kubicova L., Barbar Asskar G., Sträter N., Zahn M., Brust P., Funke U. (2010). Neue Halogenalkoxychinazoline, deren Herstellung und. Verwendung. Patent Appl..

[33] Block D., Coenen H.H., Stöcklin G. (1987). The N.C.A. nucleophilic ^18^F-fluorination of 1,N-disubstituted alkanes as fluoroalkylation agents. J. Labelled Comp. Radiopharm..

[34] Block D., Coenen H.H., Stöcklin G. N.C.A. (1988). ^18^F-fluoroalkylation of H-acidic compounds. J. Labelled Comp. Radiopharm..

[35] Waterhouse R.N. (2003). Determination of lipophilicity and its use as a predictor of blood-brain barrier penetration of molecular imaging agents. Mol. Imaging Biol..

[36] OECD (Organisation for Economic Co-operation and Development) (2004). Partition coefficient (n-octanol/water), high performance liquid chromatography (HPLC) method. OECD Guideline for Testing of Chemicals.

[37] Motulsky H.J., Christopoulos A. (2003). Fitting Models to Biological Data Using Linear and Nonlinear Regression. A Practical Guide to Curve Fitting.

[38] Pike V.W. (2009). PET radiotracers: Crossing the blood-brain barrier and surviving metabolism. Trends Pharmacol. Sci..

[39] Reinhardt R.R., Bondy C.A. (1996). Differential cellular pattern of gene expression for two distinct cGMP-inhibited cyclic nucleotide phosphodiesterases in developing and mature rat brain. Neuroscience.

[40] Seliskar M., Rozman D. (1770). Mammalian cytochromes P450—Importance of tissue specificity. Biochim. Biophys. Acta.

[41] Block D., Klatte B., Knöchel A., Beckmann R., Holm U. N.C.A. (1986). [^18^F]-labelling of aliphatic compounds in high yields via aminopolyether-supported nucleophilic substitution. J. Labelled Comp. Radiopharm..

[42] Xing D., Chen P., Keil R., Kilts C.D., Shi B., Camp V.M., Malveaux G., Ely T., Owens M.J., Votaw J. (2000). Synthesis, biodistribution, and primate imaging of fluorine-18 labeled 2b-carbo-1'-fluoro-2-propoxy-3b-(4-chlorophenyl)tropanes: Ligands for the imaging of dopamine transporters by positron emission tomography. J. Med. Chem..

[43] Höfgen N., Stange H., Schindler R., Lankau H.J., Grunwald C., Langen B., Egerland U., Tremmel P., Pangalos M.N., Marquis K.L. (2010). Discovery of imidazo[1,5-*a*]pyrido[3,2-*e*]pyrazines as a new class of phosphodiesterase 10A inhibitiors. J. Med. Chem..

